# Imaging actin organisation and dynamics in 3D

**DOI:** 10.1242/jcs.261389

**Published:** 2024-01-18

**Authors:** Thomas A. Phillips, Stefania Marcotti, Susan Cox, Maddy Parsons

**Affiliations:** ^1^ Randall Centre for Cell and Molecular Biophysics, King's College London, New Hunts House, Guys Campus, London SE1 1UL, UK; ^2^ Microscopy Innovation Centre, King's College London, Guys Campus, London SE1 1UL, UK

**Keywords:** 3D imaging, Actin, Cytoskeleton, Model organisms, Super-resolution microscopy, Bioimage analysis

## Abstract

The actin cytoskeleton plays a critical role in cell architecture and the control of fundamental processes including cell division, migration and survival. The dynamics and organisation of F-actin have been widely studied in a breadth of cell types on classical two-dimensional (2D) surfaces. Recent advances in optical microscopy have enabled interrogation of these cytoskeletal networks in cells within three-dimensional (3D) scaffolds, tissues and *in vivo*. Emerging studies indicate that the dimensionality experienced by cells has a profound impact on the structure and function of the cytoskeleton, with cells in 3D environments exhibiting cytoskeletal arrangements that differ to cells in 2D environments. However, the addition of a third (and fourth, with time) dimension leads to challenges in sample preparation, imaging and analysis, necessitating additional considerations to achieve the required signal-to-noise ratio and spatial and temporal resolution. Here, we summarise the current tools for imaging actin in a 3D context and highlight examples of the importance of this in understanding cytoskeletal biology and the challenges and opportunities in this domain.

## Teaser

**Summary:** This Review summarises the importance, challenges and approaches used for imaging and analysing actin organisation and dynamic behaviour in 3D samples.

## Introduction

Actin is a highly conserved 42 kDa molecule found in all eukaryotic organisms; it plays an essential role in the organisation of intracellular architecture of cells to enable division, shape changes, protrusion and migration. There are six actin isoforms in mammals (encoded by separate genes) that are almost identical in structure but are expressed at different levels, depending on the cellular context (Kashina, 2020). These are broadly classified into non-muscle and muscle isoforms, with diverse structures that support cell-type-specific functions. Non-muscle β-actin has been most intensively studied biochemically and is the only isoform that is critical for viability, with some redundancy shown between other isoforms (Bunnell et al., 2011). In cells, actin exists in monomeric globular (G-actin) and filamentous (F-actin) forms, with F-actin formed from multiple G-actin monomers. ATP-bound G-actin binds the barbed (+) end of F-actin and, following ATP hydrolysis, disassociates from the pointed (−) end, in a process known as treadmilling, which is critical for cellular adaptation and function (Carlier and Shekhar, 2017). This process is regulated and directed by numerous actin-binding proteins that control processes such as capping, branching and sequestration of G-actin (reviewed in Pollard, 2016). Formin proteins promote unbranched actin polymerisation via conserved formin homology (FH) domains by preventing access to capping or severing proteins, such as cofilin proteins, thus halting formation of structures such as stress fibres or filopodia (Courtemanche, 2018). The Arp2/3 complex promotes nucleation of 70° branched F-actin; this enables assembly of more-complex ‘dendritic’ architecture to support cell shape changes and new protrusions, such as those found in lamellipodia (Papalazarou and Machesky, 2021). In addition to actin nucleators, capping proteins can promote both polymerisation of branched actin filaments and prevent overextension of filament growth at the plasma membrane (Edwards et al., 2014). Actin is also found in the nucleus, in both G- and F-forms, where it contributes to chromatin architecture, transcription and survival (Hurst et al., 2019). Thus, actin dynamics and organisation crucially depend on associated proteins and are context dependent.

Much of our current understanding of F-actin organisation comes from *in vitro* studies of cells on 2D substrates, which do not recapitulate the complex environment *in vivo*. Most adherent cells, such as fibroblasts and smooth muscle cells, adopt a flat, well-spread morphology on 2D surfaces, and they assemble large F-actin stress fibres and lamellipodia. In 3D environments, these cells tend to exhibit more elongated, spindle-like organisation with fewer F-actin stress fibres compared to those in 2D environments (Caswell and Zech, 2018; Hakkinen et al., 2011). Fibroblasts can also assemble lobopodia and pseudopodia, which are blunt cortical actin or multiple extended protrusions, respectively, and are unique to 3D settings (Caswell and Zech, 2018). Variations in 3D extracellular matrix (ECM) density, stiffness or alignment can all further influence the organisation of actin (Tran and Kumar, 2021). For some cell types, such as simple epithelia or endothelial cells, growth on a 2D surface is not dramatically unlike an *in vivo* setting in terms of dimensionality. However, the mechanical properties and curvature of the 2D substrate, as well as lack of apical flow, mucus or other physiologically important cues, can result in altered morphology and F-actin organisation compared to what is seen *in vivo* (Fedi et al., 2021; Rougerie et al., 2020). This highlights the importance of studying actin assembly and the factors controlling this in more physiologically relevant 3D environments if we are to understand the biology of these processes in development, homeostasis and disease. In this Review, we will discuss tools for visualising actin organisation and the current state-of-the-art of imaging platforms in the context of 3D actin imaging. Examples of 3D actin dynamics in model organisms and *in vitro* mammalian cell-based models, such as spheroids and organoids, immune cells, fibroblasts and neurons are discussed, as are current approaches and limitations for analysing 3D actin image datasets.

## Tools for visualising actin organisation

A variety of tools exist for interrogation of actin organisation in live or fixed cells (Melak et al., 2017). Label-free methods have also been used, but do not report on actin specifically and instead rely on *a priori* or correlative dataset to define the nature of observed filamentous structures (Bon et al., 2014). For imaging fixed cells, fluorophore-conjugated phalloidin and jasplakinolide analogues are widely used owing to their highly selective binding to F-actin, fluorescent labelling and general ease of use (Pospich et al., 2020). Specific actin isoforms can be imaged with anti-actin antibodies, and G-actin using fluorescent DNase I conjugates (Patel et al., 2018). Some recently developed probes, such as silicon rhodamine (SiR; Lukinavicius et al., 2015) and SPY-conjugated actin binders (Spirochrome), can be used for both live and fixed F-actin imaging. However, as SiR and SPY dyes are conjugated to a derivative of the actin filament stabiliser and toxin jasplakinolide, dose-dependent effects on cell behaviour and viability as a result of disrupted actin function require careful consideration before use in live cells (Pospich et al., 2020). More recently, actin chromobodies (ACs) have been used to study actin dynamics in live cells. ACs are genetically encoded actin nanobodies tagged to fluorescent reporters and have enabled detailed annotation of F-actin structures, for example, those in the nucleus (Plessner et al., 2015) and associated with organelles (Schiavon et al., 2020).

Genetically encoded constructs are most commonly used to examine actin dynamics, including fusions between fluorescent proteins and actin. However, overexpressing actin itself – and introducing a bulky fluorescent tag such as GFP, even in CRISPR knock-in versions – can itself lead to artefacts and phenotypes, which can complicate interpretation (Nagasaki et al., 2017; Simiczyjew et al., 2014; Yamada et al., 2005). To circumvent this, Lifeact (a 17-residue peptide isolated from yeast) was developed; this molecule binds specifically to F-actin (Riedl et al., 2008), and has allowed live imaging of the actin cytoskeleton when conjugated to a fluorescent protein. Lifeact is now widely used for imaging actin in mammalian cell cultures and *in vivo* model organisms (Nassef et al., 2019; Riedl et al., 2008; Vidali et al., 2009), but high expression levels have also been reported to alter F-actin organisation and cell viability (Flores et al., 2019; Spracklen et al., 2014; Xu and Du, 2021). Moreover, several studies indicate that Lifeact cannot detect specific F-actin structures where its actin-binding epitope is hidden (Munsie et al., 2009; Sanders et al., 2013). Utrophin and F-tractin have also been used in a similar manner to detect F-actin structures, but have similar biases towards specific substructures within the actin molecule itself (Belin et al., 2014). Thus, a wide palette of tools is available to image actin dynamics and organisation, all with some caveats and benefits that need to be considered when designing new experiments and choosing appropriate imaging platforms.

## Platforms and considerations for imaging actin in 3D

Imaging cells in a 3D context requires several general considerations compared to imaging in 2D, such as sample depth, density and fluorescent signal. Common issues include a reduction in the signal-to-noise ratio (SNR) with imaging depth, inferior *Z*-plane resolution, and the challenge of reducing phototoxicity and bleaching, as well as loss of resolution (Fischer et al., 2011). Most of the early 2D images used to describe actin organisation were acquired using epifluorescence systems, and because cells are relatively flat, the lack of sectioning capability in these systems does not pose a problem. However, basic epifluorescence instruments are not well suited to 3D samples – or indeed those deeper than a few micrometres – as the high level of out of focus light compromises the ability to clearly resolve structural details (Bardsley et al., 2017). Point-scanning confocal microscopes offer optical sectioning that is well-suited to imaging dense actin networks, and spinning disc confocal microscopes trade off some sectioning ability (due to pinhole crosstalk) for lower illumination, and hence lower phototoxicity, imaging of dynamic processes (Fischer et al., 2011). Super-resolution by optical reassignment (SoRa) is a variant of spinning disc confocal microscopy that leverages photon reassignment to achieve super-resolved images, below the Abbe limit of ∼200 nm, without the need for more specialised sample preparation (Azuma and Kei, 2015). Similarly, DeepSIM, based on multi-spot structured illumination microscopy (SIM), and the image scanning microscopy (ISM)-inspired array of detectors can further improve resolution and reduce background (Linares et al., 2023). These approaches enable ∼100 nm resolution to be achieved in 2D samples, with an inherent reduction in both lateral and axial resolution with increasing sample depth due to sample scattering and requirement for longer lens working distances and thus the inherent reduction in resolution.

All optical microscopes use objective lenses to achieve different magnification and resolving power (the ability to distinguish between two spatially distinct points) and have variations in the objective lens working distance (WD) and numerical aperture (NA). Higher power objectives generally have a shorter WD and lower power objectives a longer WD. The colloquial ‘power’ of objectives refers to the magnification and NA, with the latter being a numerical representation of the acceptance/emission cone of photons through the objective end element. Importantly, NA also informs on the resolving power of the objective, as higher NA objectives are better able to collect high-frequency information. Higher NA (and therein resolving power) is typically achieved at the expense of shorter WD and more limited utility (Choi et al., 2018; Ross et al., 2014; Sluder and Nordberg, 2013). 3D models generally extend beyond the working distance of high-power objectives, and so lower powered 10× and 20× magnification lenses are used to compensate for sample depth, compromising on numerical aperture and inherent resolving power to achieve full volume images (Elliott, 2020). 3D samples are therefore challenging to image at higher resolution, limiting the level of detail that can be achieved.

Super-resolution methods are valuable for understanding the nanometre scale of actin organisation. Stochastic optical reconstruction microscopy (STORM) uses localisations of blinking fluorophores to generate a super-resolved reconstructed image, and stimulated emission-depletion microscopy (STED) uses a doughnut-shaped ring to stimulate emission around a sub-200 nm spot. These techniques are typically but not exclusively restricted to fixed samples; they are also often complex to use, generally can resolve to ∼10 nm resolution in 2D samples and are typically phototoxic, limiting their use for live-cell imaging. RESOLFT uses photo-switchable fluorophores to achieve sub-diffraction 3D imaging (Keller et al., 2007) but, like STED, requires non-standard hardware. Importantly in the context of imaging dynamic actin processes, RESOLFT is relatively slow due to repeated pixel-by-pixel on–off cycles, although more specialised hardware can partly circumnavigate the speed issue (Dreier et al., 2019). Expansion microscopy (ExM) adopts a different approach, and involves isometric expansion of biological samples to enable effective super-resolution imaging with standard point-scanning confocal or multi-photon systems (Chen et al., 2015). Samples can currently be expanded up to ∼20× the original sample size (Wassie et al., 2019), and this allows for effective resolution down to 15 nm, representing a considerable improvement over the ∼250 nm resolution otherwise available with diffraction-limited systems owing to the Abbe limit. ExM excels by opening 3D super-resolution scale imaging at considerable depth to groups without specialist imaging instruments, and although the process requires sample fixation, and so cannot be used to image live-cell actin dynamics, it holds significant promise for the analysis of actin organisation at high resolution in complex 3D settings.

Multiphoton instruments have historically been the gold standard for deep imaging owing to the reduction in background signal and longer wavelengths used, which reduces scattering However, higher laser power is often required to achieve sufficient signal in two-photon imaging, and the signal degrades with increasing depth (Sanderson, 2023). Adaptive optics can be introduced to correct for sample-based aberrations, such as those typically seen when imaging at depth. This can enable enhanced resolution or reduce the need for higher laser power, both of which are beneficial when imaging live 3D samples (Hampson et al., 2021; Rodriguez et al., 2021). Lattice lightsheet systems are a more recent addition to the 3D imaging toolkit, offering reduced phototoxicity and enhanced axial resolution, which is particularly valuable for imaging actin organisation in dense 3D samples (Chen et al., 2014). However, lattice lightsheet instruments typically have very small fields of view (FOV), often produce skewed images that must be computationally reconstructed and are largely restricted to non-standard sample holders, which limits throughput and ease of use. Although a small FOV can be partially circumvented by using tiling (Gao et al., 2019), the large data size of even single FOV images from most lightsheet systems remains a significant bottleneck to data handling, analysis and image presentation (Stelzer, 2021), although development of pipelines to efficiently work with these data is an ongoing process (Amat et al., 2015).

Thus, the range of instruments available for imaging actin in 3D all have their drawbacks, but ultimately, the biological question and spatial and temporal resolution needed to address this requires careful thought and planning prior to embarking on experiments. Importantly for imaging 3D actin structures, requirements for resolution, imaging speed and sample depth are crucial considerations.

## 3D actin dynamics in model organisms and tissues

Much of our knowledge of 3D subcellular actin regulation comes from work in model organisms, such as *Drosophila melanogaster* (fruit fly) (Del Signore et al., 2018), *Caenorhabditis elegans* (nematode worm) (Higuchi-Sanabria et al., 2018) and *Danio rerio* (zebrafish) (Wakayama et al., 2015). These organisms provide an inherently 3D insight within a complex context, which offers powerful perspectives not often found in mammalian cell-based models. Many model organisms benefit from short generation times and are amenable to introduction of genetic constructs encoding fluorescent proteins (FPs) of interest. A summary of model organisms and the imaging systems and probes used to study actin in 3D are shown in Table 1.

**Table 1.**
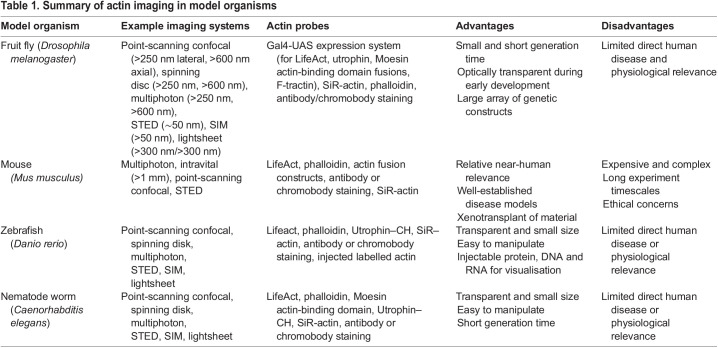
Summary of actin imaging in model organisms

The UAS/GAL4 system used in *Drosophila* model systems allows for cell- or organ-specific expression of FPs, meaning that otherwise phenotypically wild-type organisms can express mutant FPs of interest. A common approach is to generate transgenic *Drosophila* lines expressing actin-binding domains fused to FPs, such as GFP or mCherry, with actin binders, such as Lifeact, Utrophin and F-tractin (Spracklen et al., 2014). Notably, the strength of expression is important for maintaining physiologically relevant phenotypes, as strong expression of Lifeact has been shown to result in sterility and actin defects (Flores et al., 2019; Xu and Du, 2021). Expression of mutant proteins involved in actin regulation, such as members of the Arp2/3 complex and the Rho GTPases Rac1 and Cdc42, specifically in haemocytes (macrophages) coupled with LifeAct–GFP expression has revealed differing protrusion dynamics linked to these GTPases (Molina et al., 2022). Lifeact–GFP has also been employed to study polarisation of actin flow in haemocytes by leveraging particle image velocimetry (PIV) to track timelapse frame-by-frame movement (Yolland et al., 2019). *Drosophila* macrophages employ different forms of apoptotic body clearance in embryos, using Arp2/3-derived lamellipodial clearance in some cases and filopodial clearance mediated by the formins Dia and/or Ena in others, and LifeAct constructs have been used to examine colocalisation of actin with these regulators (Davidson and Wood, 2020). Another study used the GFP-tagged actin-binding domain of moesin to examine fat body cell motility and their interaction with macrophages during wound healing, and found that fat body cells migrate towards wounds in 3D using actomyosin-derived peristaltic movement, whereas formation of large lamellipodia seals off the wound site (Franz et al., 2018). In *Drosophila* oocytes, both the moesin-actin binding domain and phalloidin have been used in concert to reveal the tissue-level alignment of actin, which is maintained by egg chamber rotation (Cetera et al., 2014).

The external development of zebrafish and their relatively large size allow for manipulation at an early point in development, while their transparency makes them particularly attractive for imaging-based studies. Lifeact and Utrophin fused to the calponin homology (CH) domain (Utrophin–CH) have been used for live imaging studies in these organisms (Pinto et al., 2020) and can be expressed in specific lineages of cells, such as myocardial myofibrils, to image an otherwise normal organism and study links between, for example, ErbB2 receptor activity and actin remodelling (Reischauer et al., 2014). For instance, use of Utrophin–GFP in the developing zebrafish oocyte has revealed that there is a wave of actin polymerisation that pushes yolk granules vegetally (Shamipour et al., 2019). Actin probes can also reveal how cells interact with the surrounding microenvironment and wider tissue architecture in zebrafish under the influence of mechanical deformation (Mongera et al., 2023). Moreover, the optical clarity of developing zebrafish has allowed for development of novel super-resolution methods, such as ‘DeSOS’, which combines deconvolution with stepwise optical saturation to computationally generate super-resolution images without the need for specialist equipment (Zhang et al., 2019). DeSOS has been used in zebrafish to intravitally image distinct actin subpopulations, termed stable base clusters, and shown that there are novel structures at the neurite base; these could only be visualised upon the ∼1.5-fold improvement in lateral resolution achieved by this new technique. This highlights the versatility of this organism for understanding the fundamental changes to actin organisation during development.

*C. elegans* nematode worms are also optically transparent, amenable to expression of cell-specific FPs and have a short generation time, making it a very suitable organism in which to study 3D actin dynamics. Notably, the hermaphroditic nature of *C. elegans* makes it ideal to study reproductive organs, and many related regulatory factors are shared with mammals (Kelley and Cram, 2019). Laser ablation assays combined with PIV analysis of Lifeact–GFP have been used to demonstrate the contractile state of actin networks in *C. elegans* (Caroti et al., 2021; Saha et al., 2016). Lifeact–mKate has been used to highlight the highly dynamic F-actin rearrangement (Reymann et al., 2016) and relationship with membrane lipids in early *C. elegans* embryos (Scholze et al., 2018). *C. elegans* has well-characterised invasive behaviours, such as basement membrane invasion and leader-follower dynamics, which inherently rely on maintenance of 3D F-actin contractility (Sherwood and Plastino, 2018). Lifeact constructs have previously been used to examine invading cells in *C. elegans* (Caceres et al., 2018). In addition, expression of a moesin actin-binding domain and mCherry fusion in *C. elegans* has revealed that increasing F-actin expression and force generation, but not actomyosin contractility, for the F-actin–Arp2/3 networks means that they are sufficient to breach basement membranes in the absence of matrix metalloproteinases (MMPs) (Kelley et al., 2019).

Mouse models have also been used to study actin dynamics in 3D contexts, either through creation of transgenics or *ex vivo* labelling – with the caveat that these have slower breeding times and higher cost than the above-described model organisms. Imaging in these models is often accomplished using multiphoton microscopy, and images are generally lower resolution than for other model organisms due to deeper penetration depths and increased light scattering (Masedunskas et al., 2013) often necessitating non-standard sample preparation (Dawson et al., 2021). An invaluable facet of mouse studies is the ability to follow cell migration and invasion *in vivo* to allow corroboration of findings *in vitro*. For example, intravital single-photon confocal microscopy has revealed that inhibiting actin regulators and myosin-II prevents the transendothelial migration of lymphocytes (Yan et al., 2019), as had been previously proposed in 2D systems. *In vivo* super-resolution microscopy of actin has also been achieved using a cranial glass window and Lifeact–EYFP in cortical dendrites (Willig et al., 2014). Follow-up experiments expanded on the scope of previous work using far-red fluorophores and actin chromobodies to achieve ∼80 nm resolution and further explore dendritic morphology (Wegner et al., 2017). This superior resolution can also be achieved by extracting tissues from animals and embedding them into biomimetic environments, for example to study actin-mediated changes in stereocilia length (Drummond et al., 2015). Alternatively, tissue fixation can enable imaging of diverse actin filament arrangements, for example using expansion microscopy to reveal dense postsynaptic actin networks within mouse brain synapses (Park et al., 2020).

These model organisms provide excellent opportunities for imaging 3D actin organisation and dynamics. However, 3D cell-based model systems offer complimentary approaches to study cytoskeletal biology in human cell or tissue homeostasis and during disease evolution, as discussed below.

## 3D actin dynamics in mammalian cell-based models

**Fig. 1.**
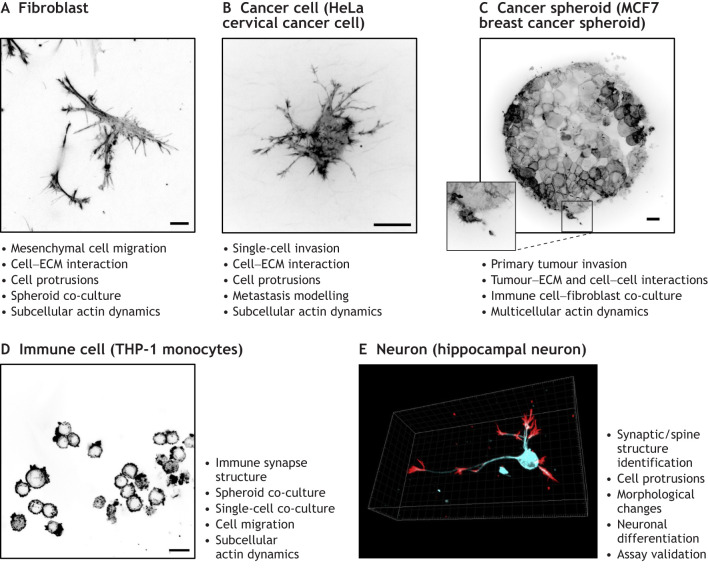
**3D actin imaging in cell-based models.** Example images of actin structures in 3D mammalian cell-based models. Maximum intensity projections of (A) fibroblasts stained with phalloidin–AlexaFluor 567, (B) collagen-embedded HeLa cell expressing mScarlet–Lifeact, (C) an MCF7 spheroid expressing mScarlet–Lifeact 24-h post-embedding in collagen with magnified inset showing finer detail of spheroid peripheral protrusions, and (D) THP-1 monocytes cultured in 3D cell-derived matrix stained with phalloidin–AlexaFluor 488. Scale bars: 20 µm. (E) Tuj 1 microtubule marker (cyan) and phalloidin (red) immunolabeled hippocampal neurons embedded in collagen fixed at 24 h. Applications and analysis parameters that can be retrieved from each cell type in 3D are indicated. Images in A–D are previously unpublished and provided courtesy of Mark-Alexander Gorey and Zhongxiao Cong (King's College London, UK). The image in E is reprinted from Santos et al. (2020) with permission from Elsevier. The cell body shown is of ∼20 μm diameter.

Encapsulation within a matrix is a common feature of many mammalian cell-based 3D models. Notably, encapsulation in matrices allows for dispersal of single cells, such as fibroblasts or cancer cells, throughout a 3D matrix in a manner that is ideally suited to imaging actin dynamics due to the proximity of the cells to the coverslip. Such proximity also opens up the repertoire of applicable imaging systems, allowing for great versatility in imaging 3D actin structures in high resolution, with confocal microscopy or super-resolution methods, or at high speed, with spinning disc confocal microscopes. Cytoskeletal organisation is known to be highly responsive to topographical and mechanical properties of the surrounding matrix, and actomyosin contractility can in turn alter matrix organisation. Fibroblasts are one of the most-studied cell types in the cytoskeletal biology field; they display a ‘mesenchymal’ morphology in 2D environments and switch to showing more cortical F-actin in 3D environments (Miron-Mendoza et al., 2017). An example image of the 3D fibroblast actin structure is shown in Fig. 1A. In 3D, actin contractility drives anterior, often branched, protrusions, which adhere to the surrounding matrix and pull the cell forward, with spinning disc confocal microscopy facilitating imaging of this dynamic behaviour (Doyle et al., 2015, 2021). These protrusions are distinct from amoeboid bodies seen in other forms of 3D migration and are often tipped with actin-rich filopodia-like structures that are distinct from those seen in 2D (Caswell and Zech, 2018).

A range of 3D cancer models offer opportunities to explore changes in actin organisation and dynamics during different stages of disease progression. Fig. 1B and C show example images of 3D actin structures for single cancer cells and spheroids embedded in collagen gels, respectively. Single cells within 3D ECM gels mimic early invasion or metastatic spread, and imaging actin in these models has enabled classification of a variety of fine-scale cellular behaviours, highlighting the versatility of such an approach for better understanding 3D actin dynamics at higher resolution. For instance, actomyosin-based cell morphologies, such as filopodia, blebbing, amoeboid and actin-rich leading edges, have been identified during invasion by imaging a 3D cell with a conventional confocal microscope (Eddy et al., 2021). Matrix-penetrating actin-rich invadopodia assembly under high confinement conditions were interrogated using Lifeact–RFP-expressing MDA-MB-231 cells embedded in collagen, again using confocal microscopy (Wisdom et al., 2018). Single-cell assays have also been used to study filopodia initiation in response to changing ECM stiffness with a powerful combination of lattice lightsheet microscopy and advanced analysis pipelines being leveraged to examine the effect of actin regulator perturbation and matrix stiffness on filopodia (Jacquemet et al., 2017; Pfisterer et al., 2020). Spheroid (cancer cell lines) and organoid (patient-derived) assemblies provide alternative means to model solid tumours and investigate actin dynamics in cancer cells as they exhibit both cell–matrix and cell–cell contacts (Fig. 1C). Super-resolution spinning disk live-cell imaging of 3D actin dynamics at the periphery of 3D tumour spheroids has been achieved by placing spheroids at defined *Z* distances from the base of an imaging chamber in spatially controlled collagen gels to allow for imaging with the SoRa microscope (Phillips et al., 2023). This helps to circumvent some of the challenges of imaging the full volume of spheroids with high magnification lenses as they can be placed closer to the base of the imaging vessel. Long-term lightsheet imaging of multiple cholangiocarcinoma organoids simultaneously revealed dynamic cell rearrangements coupled with formation of strong apical F-actin assembly at the membrane to aide in polarisation and lumen maintenance during expansion (Hof et al., 2021). Imaging the dynamics of both spheroids and organoids within physiologically relevant scaffolds is increasingly being recognised as crucial to understanding the underpinning of disease biology (Gunti et al., 2021). Rapid advances in sample preparation and imaging technology will enable such analysis to be scaled up, both in terms of resolution and sample number, with a view to using these as platforms for personalised medicine and drug discovery.

Immune cells depend on F-actin for structural support, motility and assembly of an immune synapse with antigen-presenting cells (Ritter et al., 2013). *In vitro*, immune synapses have been widely studied on 2D surfaces with immobilised antigen-coated or antigen-presenting cells (Hartzell et al., 2016). This allows for high-power objectives or super-resolution methods to image at greater resolution, which is required for imaging nanometer-scale interactions (Fritzsche et al., 2017), but at the expense of not recapitulating the *in vivo* 3D tissue environment. The structure of actin at the immune synapse depends on the specific biophysical characteristics of the ECM, with actin acting as a general marker of both cell shape and a highly dense, dynamic cortical actin structure at the synapse, as shown using live-cell confocal microscopy (Majedi et al., 2020). F-actin in migrating immune cells also plays a role in regulating nuclear deformation in dense 3D matrices, with a characteristic nuclear ‘hourglass’ deformation a common feature, both *in vitro* and *in vivo* (Thiam et al., 2016; Wolf et al., 2013; Zhao et al., 2021). Co-cultures of different combinations of immune and cancer cells have demonstrated the importance of changes in F-actin structure at the immune synapses between cell types for cytotoxic granule release and membrane deformation; conversely, increased cortical F-actin in cancer cells can result in immune cell evasion (Wurzer et al., 2019). An example of immune (monocyte) cell actin organisation in 3D collagen, demonstrating highly concentrated cortical actin with small membrane protrusions and blebs, is shown in Fig. 1D. 3D models for studying immune cells are becoming more prevalent, but their wider adoption and standardisation is still needed to determine the relevance of the observed actin dynamics and mechanisms in 2D to physiologically relevant settings (Di Modugno et al., 2019).

Actin also plays a critical role in assembly and maintenance of neuronal dendrites, axons, spines and synapses, although most studies to date have focused on 2D contexts and used electron microscopy, super-resolution fluorescence and correlative light and electron microscopy (CLEM) to study structural rearrangements. A recent study analysed differences in actin structure in both fixed and live 3D neuron cultures versus those in 2D (Santos et al., 2020; an example image of actin organisation in a single neuron in 3D from this study is shown in Fig. 1E). Interestingly, similar differences in 2D versus 3D were seen in neurons to those shown to occur in cancer cells, including reduced cell spreading and finer, longer filopodia (Santos et al., 2020). A similar 3D organisation of F-actin has been observed during neurite outgrowth and in growth cones in fixed neurons in decellularised collagen or fibronectin matrices using confocal microscopy (Sharma and Schwarzbauer, 2022). Other studies have used 3D actin imaging to validate 3D astrocyte mechanobiological assays by staining for and imaging actin to quantify actin alignment under mechanical stress (Mulvihill et al., 2018), to characterise optic nerve head astrocyte morphology and/or behaviour under the influence of TGF-β treatment (Strat et al., 2022) and to validate *in vitro* models of neuroinflammation at the blood–brain barrier by visualising neuronal cells surrounding microvasculature using multiphoton microscopy (Herland et al., 2016). These studies showcase actin imaging in 3D as a robust method for assessing neuronal cell morphology and validating experimental *in vitro* models, although many of the current approaches are limited to staining of fixed cultures. Exploring actin dynamics by using 3D live-cell imaging coupled with functional assays is thus likely to further our understanding of the underlying physiology, although a significant bottleneck in the field is the development and use of appropriate and reproducible analysis pipelines.

## Approaches to actin image analysis

To fully take advantage of optical microscopy as an experimental tool, its inherently quantitative nature should be exploited, as quantitative analysis of microscopy images can lead to unprecedented biological insights (Myers, 2012; Senft et al., 2023). All the considerations with respect to sample preparation and choosing the most suitable imaging modalities are, therefore, crucial when planning future analysis of the acquired imaging data, as all these major steps of an imaging experiment are tightly interconnected (Culley et al., 2023; Senft et al., 2023). Different analysis avenues for F-actin image analysis have been explored, covering cytoskeletal dynamics, architecture and mechanics. Most of the algorithms for the quantitative analysis of actin networks are designed for application in 2D, but some can be generalised to the third dimension. In the following section, we will initially describe the most common 2D approaches and then highlight current progress made on 3D tool development.

Characterisation of actin flow has been made possible by computationally following cytoskeletal features in time (Watanabe and Mitchison, 2002). This can be achieved by tracking either individual particles (particle tracking velocimetry, PTV) or clusters of particles within an interrogation window (particle image velocimetry, PIV); the particle displacement divided by the time spanned within subsequent frames gives an estimate of the instantaneous velocity. The choice of PTV over PIV for a given application is normally dictated by the ability of reliably segmenting single particles at an acceptable computational cost, but overall, they tend to lead to comparable results (Basak et al., 2023). Quantitative measurements of actin dynamics have led to insights in network regulation, for example, on how the motion of the leading edge in neuronal growth cone is controlled by random actin polymerisation switches (Betz et al., 2009; Wilson et al., 2010). Moreover, quantitative fluorescent speckle microscopy has enabled observations on the interdependence between actin contraction and depolymerisation (Vallotton et al., 2004). High temporal and spatial resolution live imaging of macrophages in *Drosophila* coupled with PIV-based image analyses have led to a challenge to the step-wise textbook view of cell migration, with researchers now able to propose a more holistic view of the process centred around the actin retrograde flow (Yolland et al., 2019), as well as revealing the mechanotransducive role of the physical coupling of actin networks of cells undergoing contact-mediated inhibition of locomotion (Davis et al., 2015).

Actin network architecture can inform on various physiological and pathological cell processes. One approach to characterise network organisation relies on measuring the anisotropy of the fibres it is composed of by evaluating their orientation in respect to each other and/or a reference. The computational algorithms developed for this task fall in two categories (see Marcotti et al., 2021 for a more detailed comparison). They are either based on segmentation of single fibres, such as the Fiji Ridge Detection plugin (https://imagej.net/plugins/ridge-detection), or on matrix representation or transformation of the image, such as used by OrientationJ, OrientationPy (Rezakhaniha et al., 2012) and alignment by Fourier transform (AFT) (Marcotti et al., 2021). Quantitative analyses of actin network organisation have improved our understanding of the mechanical interactions between actin and microtubules by exploiting *in vitro* essays with engineered protein crosslinkers (Preciado Lopez et al., 2014). Moreover, similar methodologies have enabled insights into the cellular response to environmental stress and substrate rigidity (Gupta et al., 2019; Gupta et al., 2015). Evaluation of actin and ECM fibre alignment has further revealed how skin fibroblasts can develop a highly aligned extracellular matrix in the presence of IL-6, suggesting potential therapeutical targets to inhibit fibrosis (Kenny et al., 2023).

The actomyosin network can be described as an active polar gel, that is, as a viscoelastic material in which polar filaments are maintained in an out-of-equilibrium state by energy consumption (Banerjee et al., 2020), as shown by various experiments using *in vitro* actin gels (Sciortino and Bausch, 2021). Active nematics, a subclass of active matter, has been widely used to model cytoskeletal fibres, leveraging on their rod-like molecules that can flow while retaining long-range orientations, similar to what is seen for liquid crystals (Marchetti et al., 2013). By exploiting this theoretical framework, it is possible to infer mechanical properties of the network by observing the dynamics of topological defects within the orientation field (Giomi et al., 2014). Similar algorithms to those used to measure network architecture described above are exploited for orientation analysis, and topological defects are detected as local minima in the metrics describing alignment order to evaluate nematics (Balasubramaniam et al., 2021; Maroudas-Sacks et al., 2021). This type of quantitative analysis has been instrumental in explaining 3D morphogenesis processes *in vivo*, where topological defects in the actin network act as structural organisational centres. For example, during the *Hydra* regeneration process, the dynamics of defects is related to the formation of the head and the foot of the animal, with fibre alignment dictating the regenerating body axis (Maroudas-Sacks et al., 2021).

**Fig. 2.**
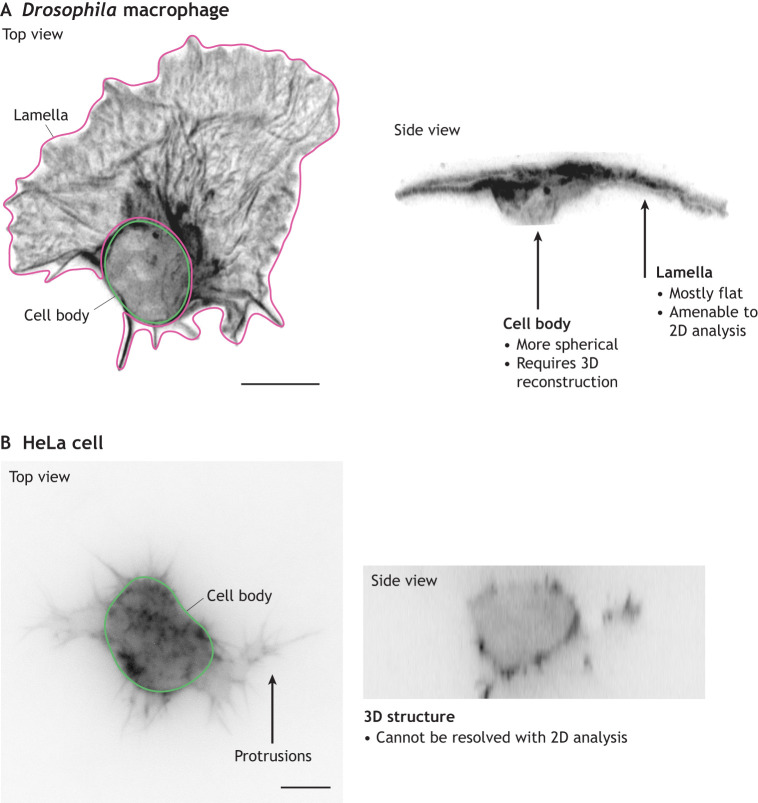
**Geometrical considerations for actin analysis.** (A) An embryonic *Drosophila* macrophage expressing Lifeact–GFP imaged live *in vivo* using confocal microscopy; shown as top view (left) and side view (right). By observing the 3D volume, it is possible to appreciate the two disparate geometries, i.e. the cell body and the lamella. Although the lamella presents a 2D flat shape amenable to 2D analysis, the cell body is spherical and would require 3D methods to define and quantify the actin structures as a volume. (B) A HeLa cell expressing Lifeact–GFP embedded within a 3D collagen gel imaged live with structured illumination microscopy; shown as top view (left) and side view (right). 3D analytical tools are required to fully capture the complex 3D morphology of this volume. Scale bars: 10 µm. Images are previously unpublished and provided courtesy of Besaiz J. Sánchez-Sánchez and Mark-Alexander Gorey (King's College London, UK).

Dedicated tools for 3D datasets are only recently emerging, and this slow progress is mostly owing to acquisition limitations (as discussed in the ‘Platforms and considerations for imaging actin in 3D’ section above) and constraints on computational resources. For instance, to perform actin flow analysis, fast temporal resolution is required, which might limit the time available at any timepoint to acquire a high number of optical slices in the *z*-axis with sufficient spatial resolution for tracking. Moreover, 3D acquisitions are often anisotropic in the *z*-axis (i.e. presenting lower spatial resolution in the *z*-axis compared to *x*- and *y*-axes), limiting the amount of information that can be extracted (Culley et al., 2023). The third dimension also adds to computational costs, both due to the requirement for more complex algorithms and the larger data file sizes. Finally, it should be noted that for some applications, the 2D acquisition and analysis is sufficient, but careful consideration of the sample geometry should be applied (Fig. 2). Fortunately, these limitations are now starting to be overcome by modern imaging modalities (e.g. lattice lightsheet imaging) and technological advancements leading to a more high-performing infrastructure (Haase et al., 2020; Ouyang et al., 2023).

A fast 3D PIV algorithm has been recently presented and applied to evaluate cell migration during *Tribolium castaneum* embryogenesis, by tracking the non-segmentable and highly dynamic actin signal during gastrulation (Pereyra et al., 2021). A novel 3D single particle tracking tool has been implemented for light-sheet datasets, for application to single molecules, adhesion complexes and larger macromolecular structures (Roudot et al., 2023). The new implementation of OrientationJ in Python (OrientationPy; https://pypi.org/project/orientationpy/) works in 3D and could also be applied to measure architectural features of 3D actin networks, by exploiting matrix representation of the image to highlight fibre anisotropy (Rezakhaniha et al., 2012). Moreover, various novel machine learning tools are being developed that are amenable to use on 3D data. These algorithms will help with processing steps that are instrumental in building a quantitative analysis pipeline, such as segmentation of actin structures (Mandal and Uhlmann, 2021; Weigert et al., 2020) and image restoration (Weigert et al., 2018), or detailed description of the cell surface morphology, shedding a light on the relationship between subcellular cytoskeletal organisation and cell shape in complex 3D environments (Driscoll et al., 2019).

## Conclusions and perspectives

The approaches used for imaging actin within 3D contexts depends on the choice of biological model system, choice of imaging systems, availability and suitability of actin probes and the most appropriate analytical pipelines that relate to the experimental goals and computational constraints. Appropriate imaging systems should be selected based on sample features and experiment requirements. Current instruments generally require sacrificing resolution, imaging speed, imaging depth, sample preparation or live imaging capability. Versatile and easy to use systems with rapid, deep, low illumination/high sensitivity and isotropic 3D imaging capabilities would be ideal for imaging actin in 3D to capture both structural details and dynamics over long time periods. Indeed, new instruments are constantly being devised with these goals in mind, but these are largely development instruments and as such are inherently not ‘turnkey’, limiting their user base until they can be commercialised. Moreover, new probes for live-cell imaging are also needed to allow for reduced perturbation of live cells typically produced by inefficient actin probe binding or due to off-target effects. Live-cell actin dyes have become more commonplace, with recent 3D live imaging of human embryonic development showcasing the use of such tools (Domingo-Muelas et al., 2023). Non-perturbing live-cell actin probes and dyes for imaging are important for samples (such as *ex vivo* tissues or primary organoids) where use of expressible probes is not possible or would influence the biology being studied (e.g. due to toxicity of transfection or increased transcriptional load). Computationally, features of 3D actin dynamics require high spatial and temporal resolution to effectively sample for fine, highly dynamic structures, generating extremely large volumes of data. Near-isotropic sampling in the axial plane and rapid imaging speed requires use of lightsheet systems that typically necessitate post-processing and generates large files that are difficult to manage (Stelzer, 2021). As many end users are not capable of storing, sharing and analysing such large datasets, the utility of such data limits new analysis approaches and represents a significant barrier in the field as recently highlighted (Eisenstein, 2023). Future efforts in the development of user-friendly and adaptable technology to increase speed, resolution and sampling depth, as well as reduce phototoxicity, will open the door to greater exploration of dynamic actin structures in a more diverse range of model organisms and 3D models or tissues. Sharing of large datasets through open access platforms and development of cloud-based tools for data interrogation will also increase a broader re-use of imaging datasets. Community-led efforts to compress and efficiently store large imaging datasets are also critical to enable experimental data to reach a broader base of computational teams for new analysis tool development and to enhance biological interpretation and understanding of actin biology in context.
